# Commentary: Suggestions for guidance by academics who collaborate with digital companies – a commentary on Bourgaize et al. (2025)

**DOI:** 10.1111/camh.70021

**Published:** 2025-07-10

**Authors:** Natalia I. Kucirkova, Todd Cherner, Adam K. Dubé, Adrian Pasquarella, Nicola Pitchford, Helen Ross

**Affiliations:** ^1^ University of Stavanger, Norway and The Open University, Stavanger, UK; ^2^ University of North Carolina Chapel Hill Chapel Hill North Carolina USA; ^3^ McGill University Montreal Quebec Canada; ^4^ University of Delaware Newark Delaware USA; ^5^ International Centre for EdTech Impact Norway; ^6^ Helen's Place Educational Consultancy UK

## Abstract

Our collective article argues for the development of a clear, shared guidance to support responsible collaborations between academic researchers and digital technology companies, particularly in the fields of education and youth mental health. Drawing on longstanding experience in edtech research, we argue that effective academia–industry collaboration requires clearer institutional support, with explicit guidance at both the contractual and community engagement levels to ensure transparency, fair reporting and the inclusion of all stakeholders. We highlight the challenges researchers face, such as limited legal support and difficulties in publishing negative results, and the need for strong contractual safeguards that protect against the suppression of negative results, define data ownership and set transparent terms for data use, publication timelines and study termination. We also advocate for formalized data‐sharing protocols and a centralized, anonymized data repository governed by shared principles, enabling more rigorous cross‐study analyses and supporting funders, researchers and policymakers in making evidence‐based decisions.

## Introduction

In their article, Bourgaize et al. (2025) propose the need for clear guidance for academics who collaborate with digital companies with the aim of enhancing youth mental health. This proposition builds on earlier responses to the critical question of whether academics should engage in such collaborations (e.g. Kucirkova, 2023; Livingstone et al., 2024).

Kucirkova (2023) argues that the issue extends beyond youth mental health to include the educational value of technologies and the role of academic collaboration with educational technology companies (edtech). Given the strong interconnection between learning and mental health (Candeias et al., 2025; Nemiro, Hijazi, O'Connell, Coetzee, & Snider, 2022), collaborative models developed by educational researchers offer valuable insights for supporting partnerships, addressing both children's learning and mental health.

We build on this argument by illustrating how academia–industry collaborations in the edtech sector have established many of the foundational elements identified as necessary by Bourgaize et al. (2025). Previous commentators have contributed to this debate, highlighting both the potential benefits and risks of academia–industry collaborations with digital companies. Here, we focus on developing guidance for responsible and effective collaborations between academic researchers (referred hereafter as researchers) and technology companies.

## The need for a shared guidance system

Research consistently demonstrates that learning outcomes are closely linked to mental health outcomes, from early childhood through adulthood (e.g. Betts & Thai, 2022). Edtech studies typically examine platforms, apps and e‐books explicitly designed for educational purposes (e.g. Weller, 2018). In contrast, research on mental health often centres on platforms primarily intended for social interaction and entertainment (e.g. Orben, Meier, Dalgleish, & Blakemore, 2024). Cross‐evaluations of these platforms are relatively rare. We argue that developing shared guidance for both fields, edtech and mental health, could facilitate such interdisciplinary assessments. This approach would enrich research insights across disciplines and promote the design of edtech products that both educate and engage users. It can also address social media platforms, which may combine entertainment with educational value.

We have collaborated with edtech companies for over 15 years across various research projects, and more recently, we have formed a virtual research group by compiling examples from our respective labs into a joint Case studies report (ICEI, 2025). These case studies illustrate that Edtech research takes diverse forms, ranging from conceptual evaluations of a tool's core features and their alignment with the learning sciences to the development of research‐based interventions designed to enhance learning outcomes. Many of these approaches are transferable to the context of youth mental health, as they address structural issues and challenges that are inherent to the digital nature of these platforms. Specifically, most digital activities examined by researchers rely on engagement data that is generated and gathered through the digital tools but is not readily available to researchers unless it is specifically requested and agreed upon for sharing. Such data is crucial for understanding the relationship between the digital tools' impact on users and needs to be addressed in the collaboration guidance.

In alignment with observations made by the UKRI‐funded *Digital Youth* programme group, we identify a notable parallel between edtech and youth mental health research: both fields lack a strong tradition of academia–industry collaboration within the humanities. This gap underscores the pressing need for structured guidance to support researchers engaging in such partnerships. Importantly, as noted by Bourgaize et al., this need spans career stages, and both early‐career and senior researchers would benefit from clearer, shared frameworks for navigating these collaborations.

Most academic institutions require ethics approval and contracts to ensure participant consent, data‐sharing terms and intellectual property protections. However, this is insufficient for addressing the more nuanced, practical questions that arise in real‐world partnerships. For instance, researchers may lack clarity on how to respond to unexpected ethical dilemmas, navigate power imbalances in partnerships or identify the appropriate institutional contact when issues emerge. The absence of detailed, scenario‐specific guidance and institutional support structures leaves many researchers underprepared, potentially compromising the integrity and impact of academia–industry collaborations in both edtech and youth mental health domains.

### Needed institutional support

To address this issue, Bourgaize et al. (2025) recommend agreeing on the purpose of the collaboration and conducting due diligence checks. Building on our own experience, we propose additional guiding principles. We argue that effective guidance is needed at two key levels:

1. Contractual level: Ensure clarity around responsibilities, publication options and reporting
2. Community engagement level: Foster transparency and mutual understanding between academic researchers, industry partners and funders of the collaborations.

Crucially, the involvement of technology users (e.g. children, parents, teachers, health professionals) should be specified during the community engagement process.

Researchers often lack adequate institutional support from their universities when engaging in novel academia–industry collaborations. A common practice for industry partners is to hire academics on short‐term consultancy contracts that outline only their hours or scope of work, but do not include detailed conditional clauses specific to collaboration agreements. When working with large corporations, researchers are frequently required to sign the company's standardized contracts, which tend to be lengthy, filled with complex legal jargon and are thus difficult for humanities researchers to fully understand. Seeking legal advice at this stage is often impractical, given the limited scope of the researcher's involvement and available resources.

### Transparent reporting of results

A critical and recurring question in these contexts concerns the handling of negative or inconclusive research results: *How should such findings be communicated and protected within these contractual frameworks?* The absence of clear contractual provisions addressing this issue can lead to conflicts over publication rights, data ownership and the ethical dissemination of findings, which, pose significant challenges to academic integrity and transparency.

Bourgaize et al. (2025) explain that principles of intellectual independence and research ethics are easier to uphold when studies yield positive results that demonstrate benefits and enhance an organization's practical impact. In contrast, reporting negative results often presents significant challenges to maintaining robust and unbiased research. Our experience suggests that the issue depends primarily on the research purpose rather than the nature of the findings themselves.

For example, when research aims to support product development and inform design improvements, negative results can be especially valuable for risk mitigation and internal learning. In such cases, companies typically prefer to keep these findings as internal reports rather than making them publicly available. Because this type of research is often funded directly by the company, it influences how the results are managed and disseminated.

Conversely, when research is intended as an independent investigative evaluation, usually funded by third parties, the ethical standard to publish all results—positive, negative and neutral—needs to be upheld and is expected by public funders. The problem is that much of the evaluation work in edtech is not supported by public funding. Public research councils tend to avoid funding evaluations of single platforms or applications, preferring projects that examine broader cross‐platform patterns or noncommercial prototypes. This approach is intended to prevent providing undue competitive advantage to specific companies through publicly funded research, but it impedes direct evaluation studies. Consequently, philanthropic organizations and private companies frequently fill the funding gap. This situation introduces many grey areas.

### Contractual safeguards

First, philanthropic funders often lack clear expectations or policies regarding the handling of negative results, while companies may actively seek to protect their brand by limiting the publication of unfavourable findings. Such practices include withdrawing from contracts without justification or prolonging data collection indefinitely in hopes of producing positive outcomes. Researchers too, especially if early in their careers, may be pushing for a publication, which is more likely to be achieved if it focuses on significant outcomes than on nonsignificant results.

Moreover, mental health and learning data are inherently variable, requiring longitudinal studies to capture meaningful patterns over time. Yet, longitudinal research often clashes with funders' and edtech's demand for rapid results. The contractual safeguards to ensure integrity in such academia–industry collaborations should include clear provisions for the transparent reporting of all results. This transparency should include expectations for timelines and conditions under which a study may be paused or terminated, ensuring that neither party can unilaterally suppress or unduly delay the publication of findings. Furthermore, contracts should include clauses that prevent companies from unilaterally terminating agreements to suppress unfavourable results, as well as provisions that limit the duration and scope of data collection to avoid indefinite extensions. Contracts must clarify data ownership and control, ensuring researchers retain the right to analyse and publish data independently. Philanthropic or other types of funders (e.g. impact investors) should mandate these conditions as prerequisites for funding approval, promoting accountability and protecting public impact from such research.

We recommend that contracts explicitly outline the various scenarios and corresponding actions through a clear, flowchart‐like framework detailing the roles and responsibilities of the researcher, company and funder. This framework should address the handling of the data sharing and analysis agreement and the establishment of a secure data pipeline and robust firewall between research and industry in the analysis and reporting of results. By managing expectations from the outset, such structured guidance facilitates the planning of analyses and publication strategies tailored to each situation and avoids scenarios where companies withhold publication rights or sponsorship of research following the data analysis stage. Moreover, these protocols should be adaptable to reflect the differing interests and stakes of each party within the collaboration, including the researcher, company and funder alike.

### Data‐sharing agreements

Our experience underscores the critical importance of formalizing contractual agreements concerning data sharing and intellectual property in writing *prior* to the commencement of any collaboration. Establishing a common protocol in the academic community for data sharing with digital companies could pave the way for creating a centralized data repository. Such a repository would aggregate anonymized data from multiple individual projects, enabling cross‐study analyses that enrich academic research with deeper insights and provide valuable benchmarks for comparison across digital companies. Such a repository may need a shared governance model where data contributors retain ownership while granting open, licensed access for collaborative data science.

### Mission creep

A separate aspect of contractual agreements relates to mission creep. This creep occurs when researchers are asked by external organisations to increase their engagement in the partnership, beyond the scope of the initial agreement, without additional funding or contractual obligations. Common requests include asking researchers to collect more data, conduct additional data analyses, disseminate to multiple audiences, meet with other organisations to report findings and secure additional funding for the external organisation. In a university funding climate based on impact, researchers can feel compelled to address the additional demands of the external organisation, to gain impact for the university and help promote their own career, often at the cost (financial and health) of the researcher.

## An online community of practice

Bourgaize et al. (2025) emphasize the importance of sharing examples of both successful and less successful collaborations to advance the field. Building on this, we stress the need to publish detailed case studies, reflective accounts and analyses of ongoing risks within the research community. Crucially, these contributions should come from both researchers and industry partners, recognizing that collaboration is inherently bidirectional. What researchers may regard as a successful partnership might differ significantly from the company's perspective and vice versa. Mutual learning from diverse experiences is essential to fostering more effective and equitable collaborations.

To further support this exchange, we propose the establishment of an online community platform that not only shares illustrative examples but also offers practical resources. These could include templates for data‐sharing agreements, sample contractual clauses and other tools designed to guide and inspire researchers and industry partners alike. Such a repository would promote transparency, facilitate best practices and help standardize approaches to common challenges. This online platform could bring together diverse stakeholders to share expertise, foster mutual learning and develop standards that enhance both research integrity and real‐world impact.

## Funding Information

Natalia Kucirkova declares no funders for her work on this manuscript. Nicola Pitchford declares no funders for her work on this manuscript. Helen Ross declares no funders for her work on this manuscript.

## Conflict of interests

Natalia Kucirkova has worked with a number of edtech organisations and investors, both as a paid consultant and on a probono basis. She is the co‐founder and director of the International Centre for EdTech Impact. Nicola Pitchford has worked with several edtech organisations and investors, both as a paid consultant and on a probono basis, in her capacity as Professor of Developmental Psychology and Head of Quality Assurance at the International Centre for EdTech Impact. Helen Ross has worked with a number of edtech organisations, as a paid consultant in her capacity as owner and founder of Helen's Place Education Consultancy.

## Ethical information

Neither informed (parental) consent nor an ethical body review applies to this paper as it does not draw on any empirical data.

## Data Availability

Data availability does not apply to this paper.
